# Clinical features and survival outcomes in IgD myeloma: a study by Asia Myeloma Network (AMN)

**DOI:** 10.1038/s41375-020-01060-w

**Published:** 2020-10-20

**Authors:** Jin Liu, Xiaoxia Hu, Yanchun Jia, Jin Lu, Jae Hoon Lee, Kihyun Kim, Wenming Chen, Aijun Liu, Yang Liu, Qi Chen, Chunyang Zhang, Cheolwon Suh, Min Kyoung Kim, Fan Zhou, Wee Joo Chng, Shaji K. Kumar, Brian Durie, Jian Hou, Weijun Fu, Juan Du

**Affiliations:** 1 Department of Hematology, Myeloma & Lymphoma Center, Shanghai Changzheng Hospital, The Second Military Medical University, Shanghai, China; 2 State Key Laboratory of Medical Genomics, Shanghai Institute of Hematology, National Research Center for Translational Medicine, Shanghai Rui Jin Hospital, Shanghai Jiao Tong University School of Medicine, Shanghai, China; 3 Peking University Institute of Hematology, Peking University People’s Hospital, Beijing, China; 4 Incheon Regional Cancer Center, Gil Medical Center, Gachon University College of Medicine, Incheon, Republic of Korea; 5 Department of Medicine, Samsung Medical Center, Sungkyunkwan University School of Medicine, Seoul, Republic of Korea; 6 Department of Hematology, Beijing Chaoyang Hospital, Capital Medical University, Beijing, China; 7 Department of Health Statistics, Second Military Medical University, Shanghai, China; 8 School of Medicine, University of Maryland Baltimore, Baltimore, Maryland USA; 9 Department of Oncology, Asan Medical Center, University of Ulsan College of Medicine, Seoul, Republic of Korea; 10 Department of Internal Medicine, Yeungnam University Medical Center, Yeungnam University College of Medicine, Daegu, Republic of Korea; 11 Department of Hematology & Oncology, Jingan District Zhabei Central Hospital Shanghai, Shanghai, China; 12 Department of Hematology-Oncology, National University Cancer Institute of Singapore, National University Health System, Singapore, Singapore; 13 Cancer Science Institute of Singapore, Singapore, Singapore; 14 Department of Medicine, Yong Loo Lin School of Medicine, National University of Singapore, Singapore, Singapore; 15 Division of Hematology, Department of Internal Medicine, Mayo Clinic, Rochester, MN USA; 16 Cedars-Sinai Medical Center, Los Angeles, CA USA; 17 Department of Hematology, Renji Hospital, Shanghai Jiao Tong University School of Medicine, Shanghai, China

**Keywords:** Myeloma, Risk factors

## To the Editor:

Immunoglobulin D (IgD) myeloma is a rare isotype that comprises 1–2% of multiple myeloma (MM) patients [1–3], which has significantly inferior survival for a median overall survival (OS) between 13 and 21 months [4–6]. Given the lack of large cohort with comprehensive clinical and cytogenetic assessment, knowledge about IgD myeloma is obtained mostly from a limited sample size [7]. Therefore, we carried out a multicenter retrospective study to evaluate the prevalence, clinical features, prognosis, and to develop and validate a prognostic model, including 356 patients with IgD myeloma from 14 centers of Asian Myeloma Network (AMN).

Data were collected from China, Korea, and Singapore diagnosed from 2002 to 2019 (Supplementary Table 1). Ethical committee approvals were obtained and study protocol was approved by the Institutional Review Board of each institution. To avoid clinical information leak, and get a real sense of the accurate model’s outcomes, we split existing 356 IgD MM to three parts, namely training cohort (one center from Shanghai, *n* = 212), validation cohort 1 (two centers from Beijing, *n* = 81), and validation cohort 2 (centers from Korea and Singapore, *n* = 63). The Least Absolute Shrinkage and Selector Operation (LASSO) Cox regression model to determine prognostic factors from the variables with *P* < 0.05 in the log-rank tests was performed as described [8, 9]. The quality of the prediction model was measured using the concordance index (C-index) and areas under the time-dependent receiver-operating characteristics (ROC) curves (AUCs). A bootstrap with 1000 re-samples was used for internal validation. SAS 9.4 and R 3.5.1 were used for the statistical analysis.

A total of 356 patients with IgD myeloma represented 2–8.8% of all myeloma patients, especially over 5% IgD myeloma prevalence in Chinese centers. We compared the clinical characteristics of IgD myeloma with 712 (1:2) non-IgD myeloma patients random selected as control matched for year of diagnosis and systemic therapy from Shanghai Changzheng Hospital. Baseline characteristics of total cohort are listed on Table 1 and different centers are shown in Supplementary Table 2. IgD myeloma patients had a higher frequency in male, younger than 65 years, advanced R-ISS stage III, hypercalcemia, elevated creatinine levels, and elevated LDH. Cytogenetic information was available for 301 patients (84.6%), while the 1q21 probe was only performed in 75.8% patients. Notably, 29.2% frequency of t(11;14) was predominantly higher compared to those in non-IgD subtypes (*P* < 0.001). Among the 88 IgD patients harbored t(11;14), the most frequent chromosome abnormalities (CA) coupled with t(11;14) were 13q- (31.8%), 1q21 + (30.7%), and followed 17p- (11.4%). ‘Double-hit’ or ‘triple-hit’ [10, 11] only occurred 5.6% and 0.3% patients, respectively.

**Table 1:** Characteristics of the study populations.

Variable	IgD MM	Non-IgD MM	*P* value
	(*N* = 356, %)	(*N* = 712, %)	
Sex			
Male	241 (67.7)	429 (60.3)	0.018
Female	115 (32.3)	283 (39.7)	
Age at diagnosis,years			
Median (range)	56 (32–85)	62 (23–96)	<0.001
<65	286 (80.3)	468 (65.7)	
≥65	70 (19.7)	244 (34.3)	
DS Stage			
I	8 (2.2)	16 (2.2)	0.812
II	23 (6.5)	54 (7.6)	
III	323 (91.3)	642 (90.2)	
ISS stage			
I	70 (19.7)	179 (25.1)	<0.001
II	64 (17.9)	262 (36.8)	
III	222 (62.4)	271 (38.1)	
Plasma cells of BM (%)			
≥50	149 (41.9)	143 (20.1)	<0.001
<50	207 (58.1)	569 (79.9)	
Hemoglobin level (g/L)			
<100	231 (64.9)	420 (59)	0.063
≥100	125 (35.1)	292 (41)	
Platelet count (10^9^/L)			
<100	76 (22.3)	91 (12.8)	<0.001
≥100	280 (78.7)	621 (87.2)	
Serum LDH (U/L)			
≥245	136 (38.2)	177 (24.9)	<0.001
<245	220 (61.8)	535 (75.1)	
Serum creatinine level (mg/dL)			
≥2	137 (38.5)	131 (18.4)	<0.001
<2	219 (61.5)	581 (81.6)	
Serum calcium level (mmol/L)			
≥2.65	85 (23.9)	105 (14.7)	<0.001
<2.65	271 (76.1)	607 (85.3)	
Light chain restriction			
Kappa	40 (11.2)	407 (57.2)	<0.001
Lambda	316 (88.8)	305 (42.8)	
Extramedullary plasmacytoma			
Yes	68 (19.1)	106 (14.9)	0.079
No	288 (80.9)	606 (85.1)	
R-ISS stage			
I	42 (11.8)	114 (16)	<0.001
II	175 (49.2)	449 (63.1)	
III	115 (32.3)	149 (20.9)	
Data missing	24 (6.7)	0 (0)	
FLCR			
0.01–100	137 (38.5)	356 (50)	0.959
≤0.01, ≥100	138 (38.8)	356 (50)	
Data missing	81 (22.7)	0 (0)	
Del (13q) in FISH			
Yes	77 (21.7)	261 (36.7)	0.001
No	224 (62.9)	451 (63.3)	
Data missing	55 (15.4)	0 (0)	
Del (17p) in FISH			
Yes	35 (9.9)	75 (10.5)	0.609
No	266 (74.7)	637 (89.5)	
Data missing	55 (15.4)	0 (0)	
1q21 gains in FISH			
Yes	91 (25.6)	368 (51.7)	<0.001
No	179 (50.2)	344 (48.3)	
Data missing	86 (24.2)	0 (0)	
t (11;14) in FISH			
Yes	88 (24.7)	96 (13.5)	<0.001
No	213 (59.9)	616 (86.5)	
Data missing	55 (15.4)	0 (0)	
t (4;14) in FISH			
Yes	4 (1.1)	136 (19.1)	<0.001
No	297 (83.5)	576 (80.9)	
Data missing	55 (15.4)	0 (0)	
t (14;16) in FISH			
Yes	3 (0.9)	8 (1.1)	0.859
No	298 (83.7)	704 (98.9)	
Data missing	55 (15.4)	0 (0)	
Double hit^a^			
yes	20 (5.6)	116 (16.3)	<0.001
no	251(70.5)	596 (83.7)	
Data missing	85 (23.9)	0(0)	
Triple hit^b^			
Yes	1 (0.3)	11 (1.5)	0.135
No	269 (75.6)	701 (98.5)	
Data missing	86 (24.1)	0 (0)	
t (11;14) and Del (13q) in FISH			
Yes	28 (7.9)	28 (3.9)	0.001
No	273 (76.7)	684 (96.1)	
Data missing	55 (15.4)	0 (0)	
t (11;14) and Del (17p) in FISH			
Yes	10 (2.8)	5 (0.7)	0.002
No	291 (81.8)	707 (99.3)	
Data missing	55 (15.4)	0 (0)	
t (11;14) and 1q21 gains in FISH			
Yes	27 (7.6)	39 (5.5)	0.012
No	243 (68.2)	673 (94.5)	
Data missing	86 (24.2)	0 (0)	
t (11;14) and t (4;14) in FISH			
Yes	1 (0.3)	0 (0)	0.124
No	300 (84.3)	712 (100)	
Data missing	55 (15.4)	0 (0)	
t (11;14) and t (14;16) in FISH			
Yes	0 (0)	0 (0)	NA
No	301 (84.6)	712 (100)	
Data missing	55 (15.4)	0 (0)	
t (11;14) and double hit in FISH			
Yes	6 (1.7)	2 (0.3)	0.003
No	265 (74.4)	710 (99.7)	
Data missing	85 (23.9)	0 (0)	
t (11;14) and triple hit in FISH			
Yes	0 (0)	0 (0)	NA
No	270 (75.8)	712 (100)	
Data missing	86 (24.2)	0 (0)	

*MM* multiple myeloma, *Ig* immunoglobulin, *DS* Durie Salmon, *ISS* international staging system, *R-ISS* revised ISS, *LDH* Lactate dehydrogenase, *BM* bone marrow, *FISH* fluorescence in situ hybridization, *Del* deletion, *FLCR* free light chains ratio, *NA* not avaliable.

^a^The cooccurrence of any 2 of the following: t(4;14), t(14;16), gain(1q), del(17p).

^b^The cooccurrence of 3 or more of the following: t(4;14), t(14;16), gain(1q), del(17p).

And then, we compared IgD myeloma patients with IgG, IgA, and light chain patients random selected as matched control (Supplementary Table 3). The median age was younger in IgD compared with others myeloma subtypes. Notably, the frequency of t(11;14) was significantly higher than non-IgD subtypes (IgD 29.2% vs IgG 10.6% vs IgA 8.4%, *P* < 0.001), but was a slight higher than light chain subtype (29.2 vs 24.9%). ‘Double-hit’ phenotype was significant lower in IgD myelomas than others subtypes.

Frontline treatment modalities used are shown in Supplementary Table 4. The overall response rate (ORR) was 88.8%, and very good partial response or better was 58.6% (Supplementary Table 5). After a median follow-up of 8.2, 7.3, and 4.9 years for the three cohorts, the median OS were 36.5 months for the total cohort and 31.2 months in training cohort, 52.2 months in validation cohort 1, and 45.7 months in validation cohort 2 (Supplementary Fig. 1**and** Supplementary Table 6). Patients received IMiDs showed a relatively longer median OS than others regimens, however, which untranslated into a significant survival benefit (*P* = 0.17, Supplementary Fig. 2a), and might be the subgroups limitation. Patients received ASCT had a median OS of 45.7 months, which was a slightly longer than 35 months for non-ASCT patients (*P* = 0.4, Supplementary Fig. 2b). We subsequently investigated whether cytogenetic aberration was a prognostic factor [12], which showed that CA did not have an impact on OS, suggesting other molecular events overcome initial CA risk features and impact prognosis.

**Fig. 1:**
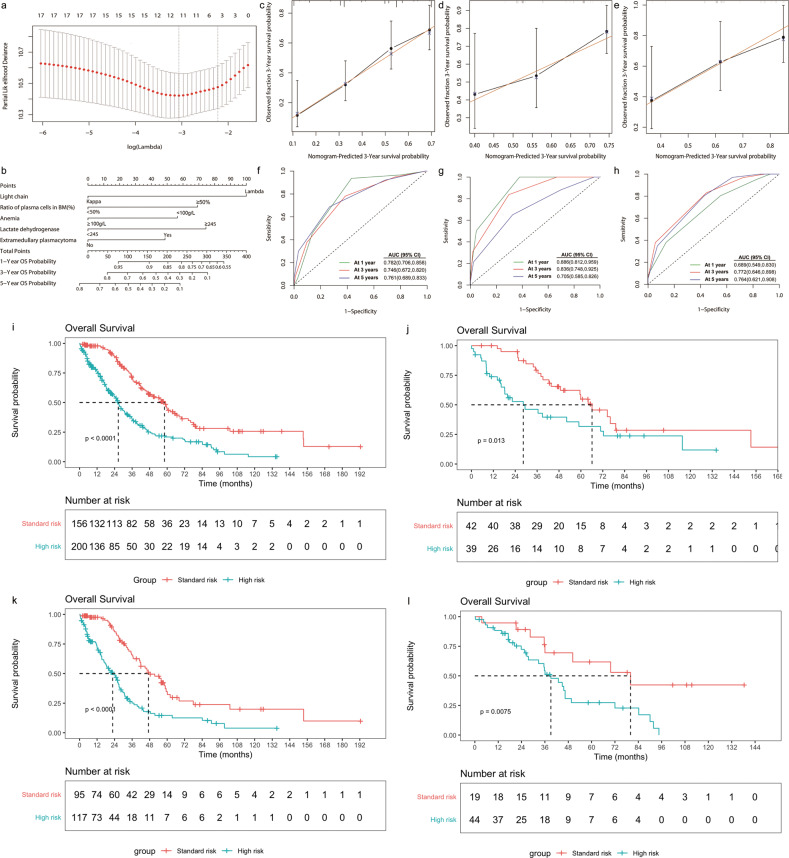
Development of a predictive model and validation. LASSO Cox regression model to determine prognostic factors from the variables with *P* < 0.05 in the log-rank tests was performed. The prediction model was established on the basis of variables selected from the LASSO Cox regression model and weighted using the Cox regression coefficient. Cross-validation for tuning parameter selection in the LASSO model (**a**). The nomogram based on data from training patients to predict individual prognosis (**b**). The calibration curves of an alternative nomogram to predict 3-year OS of IgD myeloma in training patients (**c**), validation cohort 1 (**d**) and validation cohort 2 (**e**). The *X*-axis represents the predicted survival probability calculated using the nomogram, while the *Y*-axis represents the actual survival probability for patients. The gold 45-degree line represents the ideal nomogram, while the black line represents the observed nomogram. The AUC values were 0.746–0.782 (**f**), 0.705–0.886 (**g**), and 0.689–0.772 (**h**) in the training cohort, validation cohort 1 and validation cohort 2, respectively. On the basis of the distribution of the risk scores and the 3-year survival probability, two categories of risk were created with the cut-off point at 1.56: standard risk (risk score ≤ 1.56, *n* = 156) and high-risk subgroup (risk score > 1.56, *n* = 200). The 3-year OS in patients with IgD myeloma at standard risk were significantly better than those of patients at high-risk subgroup (70.6 ± 4.1% vs 36.1 ± 3.8%, *P* < 0.0001, 1i). Similar results were obtained in the training cohort (65.8 ± 5.7% vs 25.1 ± 4.6%, *P* < 0.0001, 1k), validation cohort 1 (79.4 ± 6.5% vs 46.2 ± 8.6%, *P* = 0.013, 1j), and validation cohort 2 (69.5 ± 11.5% vs 54.4 ± 8.3%, *P* = 0.0075, 1l).

Subsequently, the LASSO Cox regression model to determine prognostic factors from the univariate analysis was performed (Supplementary Table 7). Five clinical parameters with statistically relevant, including lambda light chain, plasma cells in BM ≥ 50%, hemoglobin < 100 g/L, LDH ≥ 245 U/L, and extramedullary plasmacytoma, were integrated into multivariate LASSO regression model (Supplementary Table 8). A nomogram was developed and the risk score was computed as follows: 0.9215 × lambda light chain + 0.6376 × plasma cells in BM (≥50%) + 0.5203 × anemia (<100 g/L) + 0.6864 × LDH (≥245 U/L) + 0.4484 × extramedullary plasmacytoma (variable present = 1, absent = 0, Fig. 1a, b). The predictive accuracy for OS calculated using the C-index was 0.705 (95% CI, 0.663–0.747). In the internal validation, the corrected C-index of OS was 0.696. Similarly, the C-index for OS in validation cohort 1 was 0.690 (95% CI, 0.612–0.768) and 0.703 (95% CI, 0.608–0.798) in validation cohort 2. The calibration curves of the alternative nomogram to predict the 3-year OS presented in Fig. 1c–e suggested a good fit for the observed nomogram, when compared with the ideal nomogram. The panel displayed an AUC value at 1-year, 3-year, and 5-year OS, and the validation sets had a similar high AUC values at these timepoints (Fig. 1f–h).

On the basis of the distribution of the risk scores and the 3-year survival probability, two categories of risk were created with the cut-off point at 1.56: standard risk (risk score ≤ 1.56, *n* = 156) and high-risk subgroup (risk score > 1.56, *n* = 200). The clinical characteristics between derivations were presented in Supplementary Table 6. Patients with IgD myeloma at standard risk were significantly better than high-risk subgroup (Fig. 1i). Similar results were obtained in training and validation cohorts respectively (Fig. 1j–l). Notably we identified that ASCT showed a survival advantage in high-risk group, while it did not improve the survival in standard risk group (Supplementary Fig. 3). Moreover the prediction value of the model was independent of induction modalities (Supplementary Fig. 4).

The clinical features of IgD myeloma in this study indicated a potentially high-risk population. We identified a higher prevalence difference may be related to the difference in ethnicity, an efficient algorithm diagnosis, and assembled patients. We found 40.6% of patients had abnormal karyotypes. Translocation (11;14) was predominantly high frequency, which showed the similar to others studies [13, 14]. By far, the most clinically relevant feature of myeloma with t(11;14) is increased expression of the anti-apoptotic protein BCL-2 [15], and venetoclax of Bcl-2 inhibitor provides a new option for t(11;14), which may have a special significances for IgD patients with t(11;14).

The median OS with 36.5 months in IgD myeloma showed an inferior survival than patients with more common myeloma subtypes [16, 17]. Although the ORR (88.8%) has not improved rapidly in this AMN study, patients received IMiD with lenalidomide showed a better trend median OS than patients received PIs, conventional chemotherapy regimens, even PI + thalidomide as PI + IMiD subgroup, while PI + IMiD overcame improvement due to high peripheral neuropathy to limit the application. However, the patients received IMiD is too small to draw the definite conclusion, which might provide a clue to optimize treatment. Meanwhile, we demonstrated ASCT would benefit for IgD myeloma patients with high-risk score.

Previous studies concentrated on the prognostic value of single variable [1, 3, 5, 18, 19], as it is a challenge to construct a comprehensive prognostic model due to the rarity of IgD myeloma. We used 356 IgD myeloma patients to develop a prognostic model based on the 17 variables of the multivariate LASSO model to estimate the 3-year OS. Five clinical parameters were identified as clinically relevant to compute the risk score, which a high-risk score was consistently associated with a poor survival outcome. Moreover, the prediction value of the model was independent of induction modalities. This risk model improves the classification of IgD myeloma and may facilitate the development of risk-adapted treatment strategies. Although this retrospective design, presence of missing data, and heterogeneous treatment could have resulted in the introduction of selection bias and underestimate or overestimate of some of results, we believe that the large sample size and the multicenter study performed in such a rare disease could balance out some of these weaknesses.

In conclusion, we described the clinical features of 356 IgD myeloma patients, as well as developed and validated a predictive model containing five baseline clinical variables that could group the IgD patients into standard risk and high risk. Meanwhile, we demonstrated that IMiDs therapy might be a trend to benefit for the patient’s outcome, and ASCT could benefit patient with high risk within the predictive model. These findings may provide guidance for management of IgD myeloma and better prognostic stratification for development of risk-adapted treatment strategies.

## Supplementary information

Supplementary figure and table legends

Supplemental Fig. 1

Supplemental Fig. 2

Supplemental Fig. 3

Supplemental Fig. 4

Supplemental Table 1

Supplemental Table 2

Supplemental Table 3

Supplemental Table 4

Supplemental Table 5

Supplement Table 6

Supplement Table 7

Supplement Table 8
