# Supplementary material for: Clinical features and survival outcomes in IgD myeloma: a study by Asia Myeloma Network (AMN)
Source: Leukemia. 2020 Oct 20;35(6):1797–802. doi: 10.1038/s41375-020-01060-w (PMC8179848; doi:10.1038/s41375-020-01060-w)
Supplement: Supplementary file 1 — Supplementary figure and table legends [file 41375_2020_1060_MOESM1_ESM.docx]

**Supplemental Figure Legends**

**Supplemental Fig. 1 Survival outcomes in IgD myeloma pateints.** OS in all total cohort (a), training cohort (b), validation cohort 1 (c), and validation cohort 2 (d).

**Supplemental Fig. 2** **Survival outcomes based on the treatment regimens.** OS in patients with different induction therapy modalities (a), and ASCT or not (b).

**Supplemental Fig. 3 ASCT may overcome adverse prognosis at high risk subgroup.** ASCT showed a survival advantage over those who did not receive ASCT (3-year OS: 56.8 ± 7.5% vs. 28.4 ± 4.3%, *P* = 0.012; a), and did not improve the survival of patients in the standard risk group (b).

**Supplemental Fig. 4 Survival outcomes based on different induction therapies in high and low risk scores.** OS in patients received PIs-based (a), IMiDs-based (b), PIs +IMiDs (c), traditional alkalyating chemotherapy(d).

**Supplemental Table Legends**

**Supplemental Table 1 The distribution of patients from AMN.**

**Supplemental Table 2 Characteristics of the study populations among different distribution centers.**

**Supplemental Table 3 The baseline characteristics of IgD myeloma compared to others subtype.**

**Supplemental Table 4 Treatment modalities of the study populations.**

**Supplemental Table 5 The response to initial treatment in IgD myeloma patients.**

**Supplement Table 6 The median OS of 3 years and 5 years based on different subtypes.**

**Supplement Table 7 Effect of risk factors on OS in training cohort by univariable cox regression analysis.**

**Supplement Table 8 Effect of risk factors on OS in training cohort by LASSO logistic regression model.**
