# Supplementary figures and images for: Clinical features and survival outcomes in IgD myeloma: a study by Asia Myeloma Network (AMN)
Source: Leukemia. 2020 Oct 20;35(6):1797–802. doi: 10.1038/s41375-020-01060-w (PMC8179848; doi:10.1038/s41375-020-01060-w)

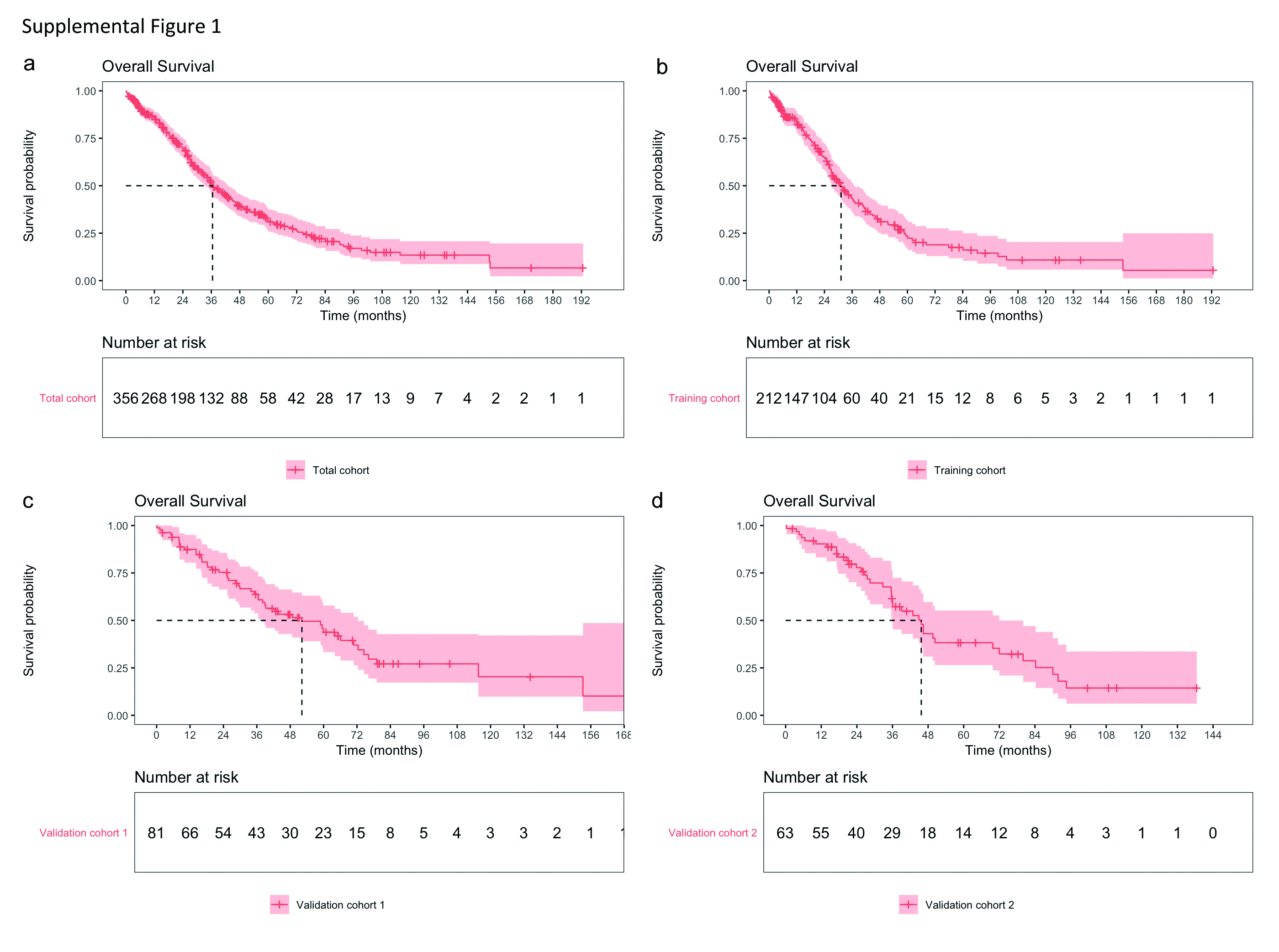

Supplement: Supplementary file 2 — Supplemental Fig. 1 [file 41375_2020_1060_MOESM2_ESM.tif]

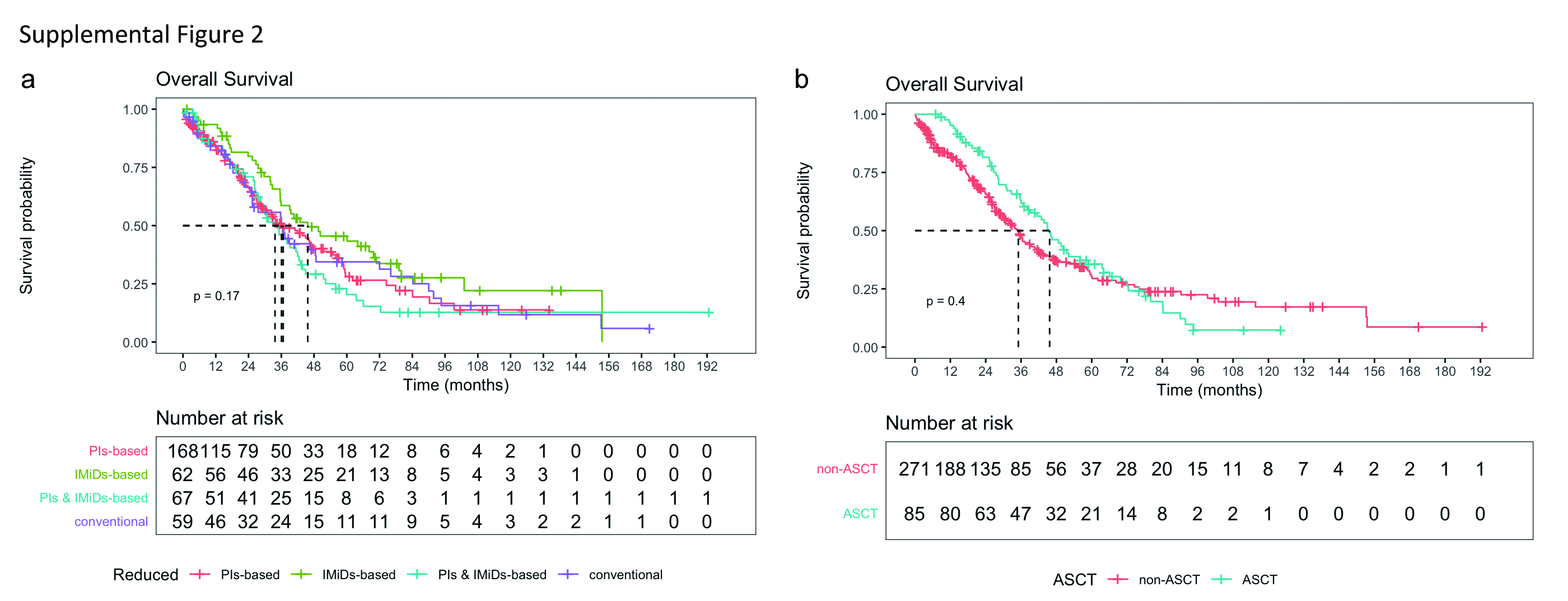

Supplement: Supplementary file 3 — Supplemental Fig. 2 [file 41375_2020_1060_MOESM3_ESM.tif]

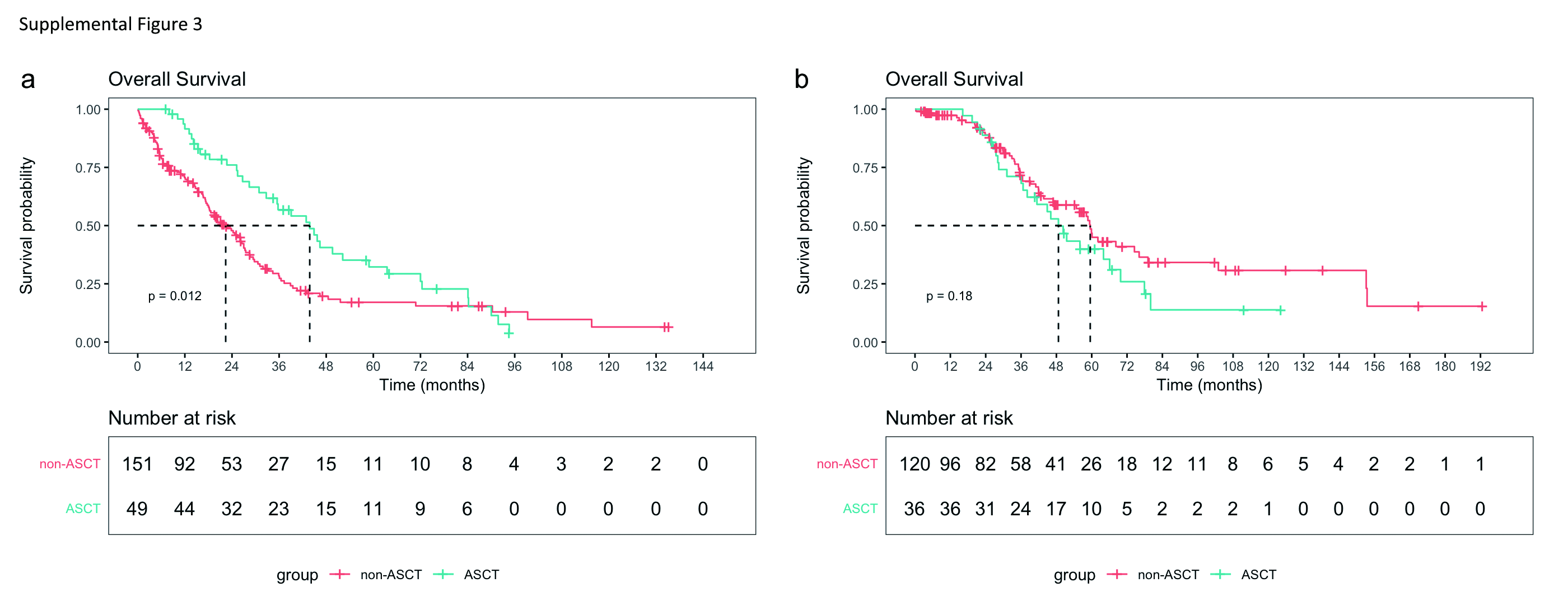

Supplement: Supplementary file 4 — Supplemental Fig. 3 [file 41375_2020_1060_MOESM4_ESM.tif]

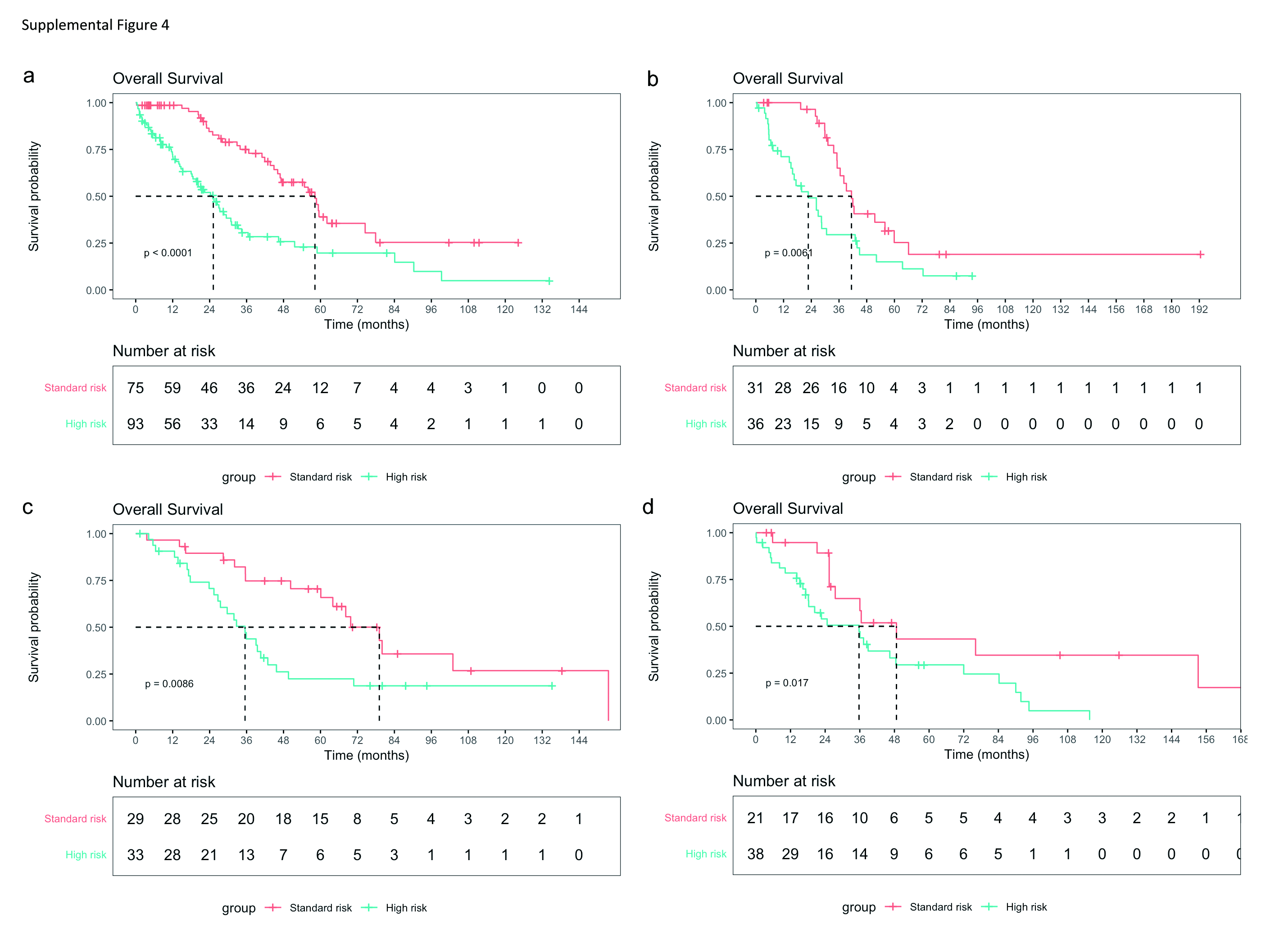

Supplement: Supplementary file 5 — Supplemental Fig. 4 [file 41375_2020_1060_MOESM5_ESM.tif]
